# Efficacy of montelukast for the treatment of pediatric allergic purpura

**DOI:** 10.1097/MD.0000000000017239

**Published:** 2019-09-27

**Authors:** Li-ping Bai, Jing Yu, Ya-xin Sun, Jiu-mei Wang

**Affiliations:** a Department of Pediatric Medicine; b Department of Endocrinology, Affiliated Hongqi Hospital of Mudanjiang Medical University; c Department of Library, Mudanjiang Medical University; d Department of Dermatology, Affiliated Hongqi Hospital of Mudanjiang Medical University, Mudanjiang, China.

**Keywords:** efficacy, montelukast, pediatric allergic purpura, safety

## Abstract

**Background:**

This study aims to evaluate the efficacy and safety of montelukast for the treatment of patients with pediatric allergic purpura (PAP).

**Methods:**

We will retrieve the following electronic databases from inception to the present: MEDILINE, Embase, CENTRAL, CINAHL, AMED, Chinese Biomedical Literature Database, China National Knowledge Infrastructure Database, Wanfang, and VIP database without language limitation. Two authors will carry out study selection, data extraction, and quality evaluation independently. RevMan V5.3 software will be used for statistical software.

**Results:**

This study will summarize high-quality evidence-based medicine to evaluate the efficacy and safety of montelukast for the treatment of PAP.

**Conclusion:**

This study will provide strong evidence to determine whether montelukast is an effective and safety treatment for PAP.

**Systematic review registration:**

PROSPERO CRD42019145472.

## 1 Introduction

Pediatric allergic purpura (PAP) is a very common hemorrhagic disease among pediatric population in clinic.^[1–3]^ It is often characterized as a small-vessel nonthrombocytopenic systemic vasculitis with the deposition of immune complexes containing immunoglobulin (Ig) A.^[4–6]^ Such disorder manifests as a palpable purpuric rash, arthralgia, gastrointestinal, and renal disorders.^[7–9]^ It has been estimated that the annual incidence of PAP is about 14 cases per 100,000 children with male to female rate of 1.2 to 1.65.^[10,11]^ Unfortunately, its etiology is still unknown.

The current clinical management of PAP mainly includes rituximab, chylothorax, triptolide, fosinopril, prednisone, vitamin E, and Chinese herbal medicine.^[12–17]^ However, up to date, there is no ideal intervention to treat this disorder. Previous studies have reported that montelukast has been widely used for the treatment of PAP.^[18–33]^ However, there is still no consistent conclusion of its efficacy for PAP. Therefore, this study will systematically explore the efficacy and safety of montelukast for treating PAP.

## 2 Methods

### 2.1 Ethics and dissemination

This study will not analyze individual data, thus no ethic approval is needed. The results of this study are expected to be published in a peer-review journal.

### 2.2 Inclusion criteria for study selection

#### 2.2.1 Type of studies

We will include randomized controlled trials (RCTs) of montelukast for the treatment of PAP. Any other types of studies, including nonclinical studies and non-RCTs, will be excluded.

#### 2.2.2 Type of participants

Any children diagnosed with PAP will be eligible for inclusion, regardless their race and gender.

#### 2.2.3 Type of interventions

Any forms of montelukast will be included in the experimental group. However, we will exclude managements of montelukast combined with other therapies.

In the control group, participants can receive any treatments, except any types of montelukast.

#### 2.2.4 Type of outcome measurements

Outcomes consist of levels of urinary beta-2 microglobulin; levels of serum IgA and IgE; disappearance time of abdominal pain, arthralgia, and purpura; quality of life (measured by any related scales, such as Quality of Life scale for Children); and adverse events.

### 2.3 Search methods for the identification of studies

#### 2.3.1 Electronic searches

We will retrieve the following electronic databases from inception to the present: MEDILINE, Embase, CENTRAL, CINAHL, AMED, Chinese Biomedical Literature Database, China National Knowledge Infrastructure Database, Wanfang, and VIP database without language limitation. The detailed strategy for searching the MEDILINE database is shown in Table 1. Equivalent search strategy will be adapted in other electronic databases.

**Table 1:**
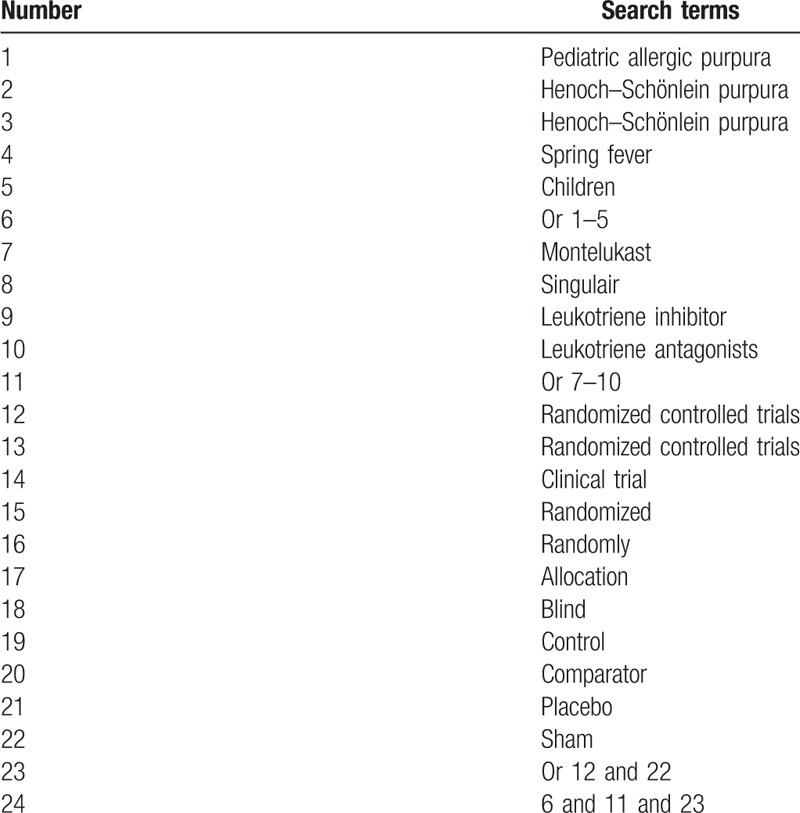
Search strategy used in MEDLINE database.

#### 2.3.2 Other resources

Literature records, such as conference proceedings, dissertations, research projects, and reference lists of relevant reviews will also be searched.

### 2.4 Data collection and analysis

#### 2.4.1 Selection of studies

Prior to the study selection, an eligibility criteria and later procedures will be built by all authors through discussion. All literature records will be confirmed by 2 independent authors based on the previous designed eligibility criteria. Any discrepancies between 2 authors will be solved by consensus with the help of a 3rd author. Authors will read the titles and abstracts of all search records to select studies suitable for inclusion. Then, all irrelevant and duplicated literature will be excluded. After that, all remaining studies will be read by full-texts to check if they meet the final inclusion criteria. The procedure of study selection will be presented in the flow diagram with specific reason for each excluded study at different stages.

#### 2.4.2 Data collection and management

Before data extraction, a standard data collection sheet will be created after discussion among all authors. Two independent authors will collect data using such previous designed form. If 2 authors have different views, this issue will be solved through consultation with a 3rd author. The collected items consist of title, 1st author, publication time, region, patient characteristics, study setting, sample size, study methods, details of interventions in the experimental and control group, outcome measurements, adverse events, and any other detailed information.

#### 2.4.3 Risk of bias assessment

Two authors will independently evaluate seven domains of bias using Cochrane risk of bias tool. Each domain will be further classified into 3 levels of bias: high, unclear, and low risk of bias. Any differences regarding the risk of bias assessment between 2 authors will be solved by discussion or consultation with a 3rd independent author in this study.

#### 2.4.4 Treatment effect measurement

Discontinuous outcome data will be calculated with risk ratio with 95% confidence intervals. Continuous outcome data will be calculated with mean difference or standardized mean difference with 95% confidence intervals.

#### 2.4.5 Dealing with missing data

The 1st authors will be contacted by email or telephone to require the missing data if possible. If that data cannot be inquired, we will analyze the available data and will discuss its potential influence in the discussion section.

#### 2.4.6 Assessment of heterogeneity

Higgins *I*^2^ test will be utilized to test inconsistencies among eligible studies. The cutoff point for *I*^2^ test is 50%. *I*^2^ ≤ 50% indicates acceptable heterogeneity among studies. *I*^2^ > 50% means the existence of substantial heterogeneity among studies.

#### 2.4.7 Assessment of reporting biases

If more than 10 eligible RCTs will be included in this study, potential reporting bias will be investigated by funnel plot^[34]^ and Egger regression test.^[35]^

#### 2.4.8 Data synthesis and analysis

RevMan V5.3 software will be utilized for data synthesis and meta-analysis. If *I*^2^ ≤ 50%, a fixed-effect model will be used for data synthesizing, and meta-analysis will be carried out if the same outcome of sufficient studies are included. If *I*^2^ > 50%, a random-effects model will be applied for data pooling, and subgroup analysis will be conducted. If there is still significant heterogeneity after subgroup analysis, we will not perform meta-analysis. Instead, we will report outcome results as a narrative summary.

#### 2.4.9 Subgroup analysis

Subgroup analysis will be performed to identify possible reasons for significant heterogeneity based on the different location, treatments, comparators, and outcomes.

#### 2.4.10 Sensitivity analysis

When sufficient data are available, sensitivity analysis will be carried out to explore the stability of pooled outcome results after eliminating low-quality studies.

## 3 Discussion

Due to the increasing incidence of PAP among the pediatric population, and consequently the rise in need for the management, it is very essential for both clinicians and patients to explore an effective therapy with satisfied safety profile such as montelukast. However, there is still lack of evidence-based medicine support regarding the efficacy and safety of montelukast for children with PAP. Through this study, we hope to eliminate this gap, and will offer an up-to-date evidence of montelukast for the treatment of PAP. In the event of inconclusive evidence, this study will explore an area for both clinical practice and further research. Thus, we believe this study is very helpful for children who have PAP and are keen to try montelukast therapy.

## Author contributions

**Conceptualization:** Ya-xin Sun, Jiu-mei Wang.

**Data curation:** Jing Yu, Jiu-mei Wang.

**Formal analysis:** Jing Yu, Ya-xin Sun.

**Funding acquisition:** Jiu-mei Wang.

**Investigation:** Jiu-mei Wang.

**Methodology:** Jing Yu, Ya-xin Sun.

**Project administration:** Jiu-mei Wang.

**Resources:** Jing Yu, Ya-xin Sun.

**Software:** Jing Yu, Ya-xin Sun.

**Supervision:** Jiu-mei Wang.

**Validation:** Jing Yu, Ya-xin Sun, Jiu-mei Wang.

**Visualization:** Jing Yu, Ya-xin Sun, Jiu-mei Wang.

**Writing – original draft:** Jing Yu, Ya-xin Sun, Jiu-mei Wang.

**Writing – review & editing:** Jing Yu, Ya-xin Sun, Jiu-mei Wang.
